# Land Snails as a Valuable Source of Fatty Acids: A Multivariate Statistical Approach

**DOI:** 10.3390/foods8120676

**Published:** 2019-12-12

**Authors:** Francesco Giuseppe Galluzzo, Gaetano Cammilleri, Alessandro Ulrici, Rosalba Calvini, Andrea Pulvirenti, Giovanni Lo Cascio, Andrea Macaluso, Antonio Vella, Nicola Cicero, Antonella Amato, Vincenzo Ferrantelli

**Affiliations:** 1 Istituto Zooprofilattico Sperimentale della Sicilia, via Gino Marinuzzi 3, 90129 Palermo, Italy; francescogiuseppe92@gmail.com (F.G.G.); giovanni.locascio71@gmail.com (G.L.C.); andrea.macaluso@izssicilia.it (A.M.); laboratorio.residui@gmail.com (A.V.); vincenzo.ferrantelli@izssicilia.it (V.F.); 2 Dipartimento di Scienze della Vita, Università degli studi di Modena e Reggio Emilia, Via Università 4, 41121 Modena, Italy; alessandro.ulrici@unimore.it (A.U.); rosalba.calvini@unimore.it (R.C.); andrea.pulvirenti@unimore.it (A.P.); 3 Dipartimento SASTAS, Università degli studi di Messina, Polo Universitario dell’Annunziata, 98168 Messina, Italy; ncicero@unime.it; 4 Dipartimento di Scienze e Tecnologie Biologiche Chimiche e Farmaceutiche, Università degli Studi di Palermo, Viale delle Scienze, 90128 Palermo, Italy; antonella.amato@unipa.it

**Keywords:** fatty acids, land snails, GC-FID, heat processing, principal component analysis

## Abstract

The fatty acid (FA) profile of wild *Theba pisana*, *Cornu aspersum,* and *Eobania vermiculata* land snail samples, collected in Sicily (Southern Italy), before and after heat treatment at +100 °C were examined by gas chromatography with a flame ionization detector (GC-FID). The results show a higher content of polyunsaturated fatty acids (PUFAs) in all of the examined raw snails samples, representing up to 48.10% of the total fatty acids contents, followed by monounsaturated fatty acids (MUFAs). The thermal processing of the snail samples examined determined an overall reduction of PUFA levels (8.13%, 7.75%, and 4.62% for *T. pisana*, *C. aspersum* and *E. vermiculata* samples, respectively) and a species-specific variation of saturated fatty acid (SFA) contents. Oleic acid remained the most abundant FA of all of the snails species examined, accounting for up to 29.95% of the total FA content. A relevant decrease of ɷ3/ɷ6 ratio was found only for *T. pisana* samples. The principal component analysis (PCA) showed a separation of the snail samples in terms of species and heat treatment. The results of this work suggest land snails as a valuable source of MUFA and PUFA contents and boiling as appropriate treatment, according to the maintenance of healthy properties.

## 1. Introduction

Terrestrial gastropods, commonly named land snails, constitute a niche food product traditionally appreciated by many European countries, especially France and Italy. The use of land snails as food is still steadily growing, and 26,000 tons of snails were imported from Africa and countries in the Middle East [1]. *Cornu aspersum*, *Eobania vermiculata,* and *Theba pisana* are the land snail species most consumed in Italy [2]. Land snails are consumed in different ways all over the world, but the principal cooking procedures recognized are roasting and boiling, according to the traditions of the countries. According to Milinsk et al. [3], there is a correlation between land snails’ diet and their nutritional values. Recently, increasing attention was paid to the fatty acid composition, due to nutritional and health-related aspects [4,5,6,7,8]. However, few studies are available about the fatty acid (FA) profile in land snails [3,9,10] and, as far as we know, no data have been reported regarding the fatty acid profile of *T. pisana*. Snails are commonly consumed in different ways after boiling due to the risk posed by the possible presence of potentially pathogenic microorganisms [2]. The cooking temperature can influence the nutritional aspect of mollusks [11]. At present, there are too few studies about the influence of heat processing (such as boiling) on the nutritional composition of land snails.

In this context, the present work aimed at evaluating the fatty acids content of wild *C. apsersum*, *T. pisana,* and *E. vermiculata* samples collected in Sicily (Southern Italy). Furthermore, the effect of boiling on the fatty acid composition was evaluated to have a comprehensive nutritional evaluation of this product after processing.

## 2. Materials and Methods

### 2.1. Reagents and Standards

All chemicals, solvents, and reagents employed were of analytical grade (≥99.9%). Acetone, hexane, diatomaceous earth, sodium sulfide nonahydrate, methanol, and hydrochloric acid were purchased from Sigma-Aldrich (Amsterdam, Holland). All of the gas used for gas chromatography (GC) analysis was pure (≥99.9995%). Water used for the separation of fatty acid methyl esters (FAMEs) phase was bidistilled in Milli-Q^®^ Integral 5 (Merck KGaA, Darmstadt, Germany). FA standards were purchased from Sigma-Aldrich (Amsterdam, Holland). The 10,000 mg/L standards were prepared by diluting 100 mg of a pure standard solution with 10 ml of n-hexane. A mixture of FA standards was used for the identification of each peak.

### 2.2. Sample Collection and Preparation

**Figure 1:**
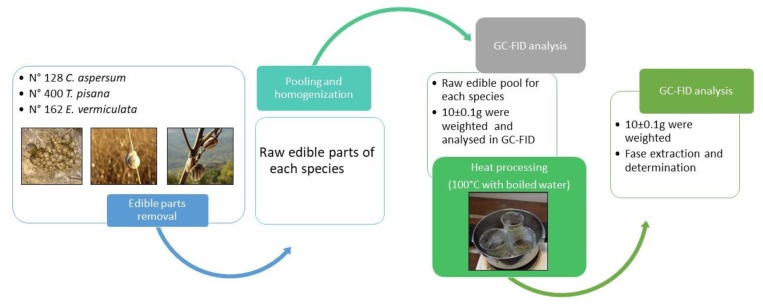
Scheme of the cooking process of the land snails samples collected.

A total of 128 samples of *C. aspersum*, 400 samples of *T. pisana,* and 162 samples of *E. vermiculata*, were collected from Palermo provinces (Sicily, Southern Italy) in 2018 during July for *C. aspersum*, August for *T. pisana*, and September for *E. vermiculata* to have the maximum assimilation efficiency according to the literature [12,13,14]. The shell of the snail samples was removed and only the meat was considered for the chemical analysis. The meat of the snail samples was grouped into three pools according to the species, then homogenized by a vertical mixer B-400 (Büchi, Flawil, Switzerland) and stored at −10 °C for 24 h to prevent a decrease in fatty acid content during the storage period [15]. The FA content of each sample pool was determined both raw and after cooking at 100 °C with boiled water for 30 s. The entire procedure of analysis is shown in Figure 1.

### 2.3. Extraction of Fatty Acids and Gas Chromatography with a Flame Ionization Detector (GC-FID) Analysis

An amount of 10 ± 0.1 g of each pool of samples was placed in a glass of polypropylene and mixed with diatomaceous earth (Sigma-Aldrich, Amsterdam, Holland). The mixture was transferred in an accelerated solvent extraction (ASE) ASE 200 cell (Thermo Fisher, Waltham, Massachusetts, USA). The ASE operating conditions were set up as follows: 20 mL of hexane/acetone, 70:30; extraction temperature 120 °C for 6 min with a pressure of 120 pound per square (PSI).

The extract was filtered (size 240 nm) and dehydrated in rotavapor (Büchi, Flawil, Switzerland) at +40 °C. For the preparation of FAME, 100 mg of the oil extracted was trans-esterified in a pyrex tube by using 2 mL of HCl/MeOH (2:98 v/v) to obtain the fatty acid methyl esters (FAMEs). The solution was mixed in a vortex for 1 min and put in the oven at 120 °C for 1 h. After cooling, 2 mL of bidistilled water and 1 mL of hexane were added, and the mixture was centrifuged at 300 rpm for 1 min. Approximately 1 mL of the upper n-hexane phase was transferred in a vial and injected in gas chromatography (GC) with a flame ionization detector (FID).

Each pool of samples was examined in triplicate by GC-FID analysis. The analysis was carried out by a Trace GC/ULTRA HP 5890 GC + 7673 A/S (Thermo Fisher, Waltham, Massachusetts, USA); a Famexax column (30m × 0.25 mm i.d. × 0.25 µm df) was used for the separation. A flame ionization detector (FID) and ChromQuest 4.2.1 software (Thermo Fisher Scientific, Waltham, Massachusetts, USA)were used for the qualification and quantification of the analytes. The injector port and the detector temperatures were 220 °C and 230 °C, respectively. The split ratio was 1:20. The flow rates of compressed air and hydrogen were 350 mL min^−1^ and 35 mL min^–1^, respectively. The carrier gas was helium (1.5 mL min^−1^). The oven temperature was programmed at a rate of 6.0 °C min^−1^ from 130 to 225 °C, held for 15 s.

Individual FAME was identified by comparison with the chromatographic behavior of authentic standards by the formula:

(1) $$TR=TR{\;}_{st}\pm 0.5$$

where *TR* is the determined retention time (min), and $TR{\;}_{st}$ is the retention time for each FA standard. The relative percentages of the fatty acids were also determined. Quantitation of individual FAs is thus based on the comparison of their peak areas (Ai), and the peak area of a suitable standard. The relative percentages of fatty acids (C) were determined by the formula:

(2) $$C=\frac{A}{\sum^{\;}A}100$$

### 2.4. Validation of the GC-FID Method

The repeatability mean and standard deviation of the analytical procedure were all calculated according to Taverniers et al. [16]. Separated FA standards were used to calculate the mean retention times (RTs) in the FID detector. The precision of the quantitative method was checked by the repeatability test, based on ten series of experiments [17]. The area of each peak was measured and corrected manually. The relative percentage of the fatty acids was also determined by comparing their peak areas.

### 2.5. Data Collection and Statistical Analysis

The data were expressed as g/100g FA in fat extracted and grouped according to species and treatment (raw *T. pisana, C. aspersum,* and *E. vermiculata* and cooked *T. pisana*, *C. aspersum,* and *E. vermiculata*). The variation of fatty acids after heat treatment was calculated as follow:

(3) $$Fa_{\%}=100-\left(\frac{Fa_{b}}{Fa_{raw}}\right)100$$

where $Fa_{\%}$ is the variation (expressed as a percentage), $Fa_{b}$ and $Fa_{raw}$ are the fatty acid content in boiled and raw samples, respectively (expressed as mg/100 g).

Before calculating the principal component analysis (PCA) model, the erucic acid variable was removed from statistical analysis because its presence was found only in raw *T. pisana* samples. All of the variables were pre-treated by Pareto scaling [18,19], in order to have a compromise between highlighting the contribution of the most abundant analytes and keeping at the same time the information brought by the less abundant ones. The PCA model was calculated using the software PLS-Toolbox ver. 8.6 (Eigenvector Research Inc., Wenatchee, WA, USA), running in the MATLAB environment (ver. 9.3, The Mathworks Inc., Natick, MA, USA).

## 3. Results

### 3.1. Fatty Acid Profiles

The fatty acid contents of the land snail samples examined are shown in Table 1. Seventeen FAs were found in all of the species examined: Six saturated fatty acids (C_14:0_, C_16:0_, C_17:0_, C_18:0_, C_20:0_, C_22:0_), six monounsaturated (C_14:1_, C_16:1_, C_17:1_, C_20:1_, C_18:1ɷ:9_), and six polyunsaturated (C_18:2ɷ:6_, C_18:3ɷ:3_, C_20:2_, C_20:4_, C_20:5_, C_22:6_). Erucic acid (C_22:1_) was found only in raw *T. pisana* samples.

### 3.2. Fatty Acids of Raw Samples

The polyunsaturated fatty acid (PUFA) content of raw *T. pisana*, *C. aspersum,* and *E. vermiculata* was 48.10 g/100 g, 47.22 g/100 g, and 46.56 g/100 g, respectively, representing the most abundant class of fatty acids, followed by monounsaturated fatty acid (MUFA) in *T. pisana* and *E. vermiculata* (31.27 g/100 g and 27.86 g/100 g, respectively) and saturated fatty acid (SFA) in *C. aspersum* (26.97 g/100 g).

The main PUFA components were C18:2ω6 (18.78–22.15 g/100 g), C18:3ω3 (7.64–15.78 g/100 g), and C20:2 (3.98–5.29 g/100 g). Linoleic acid (C18:2 ω6) represents the most abundant PUFA showing a range between 7.64 and 15.78 g/100 g. A high level of eicosapentaenoic acid (C20:5) was determined in *T. pisana* samples (9.85 g/100 g).

The MUFA profiles obtained for all the species examined consisted of C14:1 (0.23–0.53 g/100 g), C16:1 (0.32–0.5 g/100 g), C17:1 (0.52–1.01 g/100 g), C18:1ω:9 (23.79–28.83 g/100 g), and C20:1 (0.17–0.37 g/100 g). Oleic acid was the main component of all the samples examined, representing 25% of the total fatty acid content. Erucic acid was found only in raw *T. pisana* samples at low concentrations (0.52 g/100 g).

Among the SFA, palmitic acid (C16:0) was the most abundant in all of the samples examined (from 12.63 to 16.02 g/100 g), followed by stearic acid (5.41–7.66 g/100 g). The SFA profiles in all of the species consisted of C14:0 (ranging from 0.73 to 0.81 g/100 g), C16:0 (12.63–16.02 g/100 g), C17:0 (1.02–1.36 g/100 g), C18:0 (5.41–7.72 g/100 g), C20:0 (0.63–0.81 g/100 g), and C22:0 (0.31–0.64 g/100 g).

The raw *T. pisana* samples showed the highest ω3/ω6 ratio (0.58), followed by *E. vermiculata* (0.55) and *C. aspersum* (0.43).

### 3.3. Fatty Acids after Heat Treatment

After boiling at +100 °C, the FA profile of all of the species examined verified a decrease of PUFA content up to 8.2%. Differently from PUFA, a species-specific modification of MUFA and SFA contents was found.

In particular, *T. pisana* samples showed an increase of SFA content by 22.20%; only C22:0 verified a decrease after boiling (from 0.31 to 0.29 g/100 g).

The MUFA contents decreased by 2.49%. No erucic acid was found after boiling. The PUFA content decreased from 48.10 to 44.19 g/100 g (8.13%), with a significant reduction of C20:5 content (from 9.85 to 1.15 g/100 g).

Regarding *C. aspersum*, the SFA content decreased by 14.68%, showing a reduction of C20:0 from 0.71 to 0.39 g/100 g; only C17:0 showed an increase from 1.13 to 1.35 g/100 g.

The total MUFA content increased by 29%. Palmitoleic acid (C16:1) showed a significant increase from 0.32 to 1.35 g/100 g, followed by C14:1, C17:1, and C30:1.

The *E. vermiculata* samples verified a reduction of SFA content after boiling (8.72%), especially for arachidic acid (C20:0) (from 0.81 to 0.37 g/100 g), followed by C17:0 (1.36–1.04 g/100 g) and C14:0 (0.81–0.63 g/100 g). The behenic acid (C22:0) content increased by 25.81%. The MUFA content verified an increase from 27.86 to 32.24 g/100 g. Palmitoleic acid (C16:1) was the only MUFA that decreased, from 0.40 to 0.37 g/100 g. The heat treatment of the *E. vermiculata* samples determined a decrease of PUFA components of 4.61%. Eicosapentaenoic acid (C22:6) decreased significantly from 1.30 to 0.34 g/100 g. The MUFA fatty acids more compromised after boiling were palmitoleic acid (C16:1) for *E. vermiculata* and *T. pisana* samples and myristoleic acid (C14:1) for *C. aspersum*. Oleic acid (C18:1ɷ9) remained the main component of all the land snails species examined even after boiling. Moreover, no oleic acid content variation was found after boiling for *T. pisana* samples.

Among the PUFA group, eicosapentaenoic acid (C20:5) showed the highest decrease in *T. pisana* and *E. vermiculata* samples after the heat treatment, whereas docosahexaenoic acid (C22:6) decreased up to 70% in *C. aspersum*.

A decrease of the ω3/ω6 ratio was found only for the *T. pisana* samples, whereas the *E. vermiculata* and *C. aspersum* samples increased the ω3/ω6 ratio up to 35%. Finally, a reduction of the PUFA/SFA ratio was found only for *T. pisana* samples.

### 3.4. Multivariate Analysis

**Figure 2:**
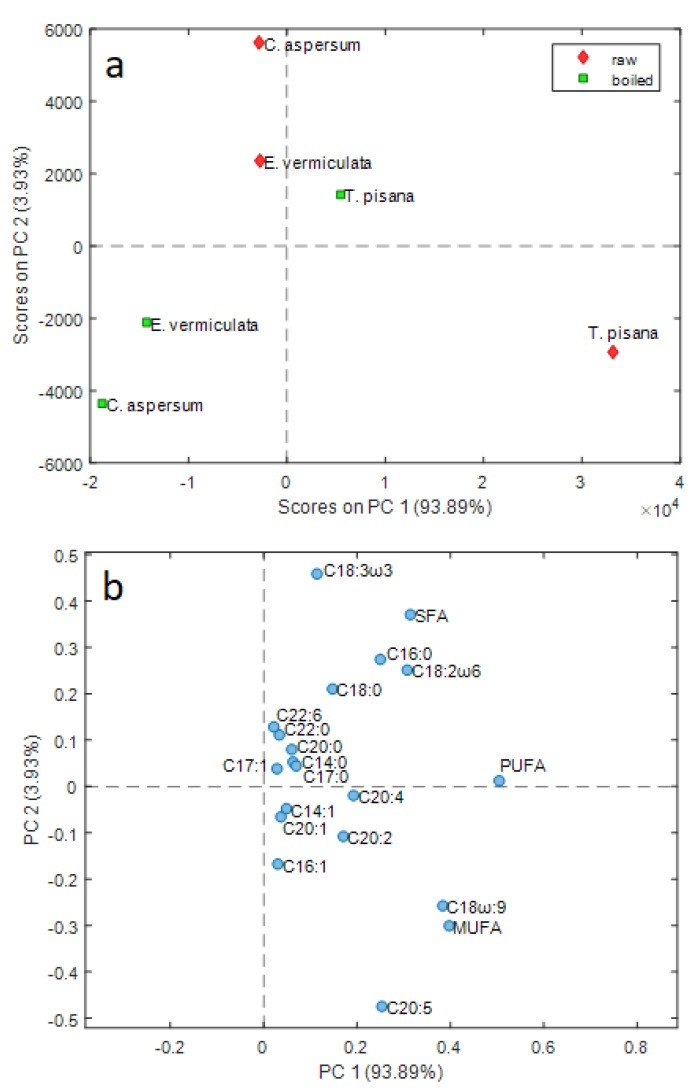
PC1 vs. PC2 score (**a**) and loading (**b**) plots of FA contents of the land snail samples examined, according to the species and treatment (raw vs. boiled).

Given the high number of fatty acids examined as variables, principal component analysis (PCA) was used to explore the dataset structure and to obtain more information on the variables that mainly influence sample similarities and differences after heat treatment. The PCA model calculated after Pareto scaling showed that the data group variation is visible in the first two principal components, accounting for 97.82% of total data variance. Figure 2, which reports the PC1 vs. PC2 score plot (Figure 2a), together with the corresponding loading plot, highlights that PC1 alone explains about 94% of data variance. This extremely high value, together with the fact that all the variables have positive loading values along PC1 (Figure 2b), reflect the high positive correlation among the most significant part of the considered variables, so PC1 describes the prevailing trend of the analyzed FAs for the samples examined.

The score plot shows differences related both to species and to heat treatment. PC1 describes a decrease in the number of fatty acids for each species after boiling. The variation is more marked for the *T. pisana* samples, which in general show the highest amount of unsaturated fatty acids (UFAs). In fact, both raw and boiled *T. pisana* samples lie at positive values of PC1, whereas all *E. vermiculata* and *C. aspersum* samples lie at negative values of PC1; this is due to the fact that, notwithstanding the significant decrease of FA content, boiled *T. pisana* still has a UFA content higher than raw *E. vermiculata* and raw *C. aspersum*. These last two samples have essentially the same overall amount of FAs and boiling leads to a more marked decrease for *C. aspersum* than for *E. vermiculata*.

*T. pisana* shows an opposite trend compared to the two other species considering the position along PC2 of the samples before and after boiling. Recalling that the percentage of variance explained by PC2 (3.93%) is much lower than the percentage of variance explained by PC1 (93.89%), PC2 accounts for the differences between the different species in the variations of the FA compositions after boiling, beyond the overall decrease, explained by PC1.

These variations of the FA profile can be examined more in-depth by means of the corresponding loading plot reported in Figure 2b. In this plot, the variables that are far from the origin contributed to the systematic variability explained by the PCA model, whereas variables close to the origin (like, e.g., C17:1, C14:0, C20:2, C20:1, C22:6, etc.) did not show a systematic trend.

The significant variations after boiling for all of the three species examined are related to PUFA, MUFA, oleic acid, SFA, and linoleic acid, which show higher PC1 values. PC2 showed a different variation of the fatty acid contents between the *T. pisana* samples and the other two species. A more considerable variation of eicosapentaenoic (C20:5) acid, MUFA, and oleic acid was found for *T. pisana* samples, whereas *E. vermiculata* and *C. aspersum* showed a more significant variation of SFA, linoleic acid, palmitic acid, and linolenic acid.

## 4. Discussion

Fatty acids are ubiquitous molecules in biological systems. They play several roles in metabolism, as structural components in membrane lipids, and as precursors of some molecules like prostaglandins and eicosanoids [20]. The dietary intake in favor of PUFA and MUFA instead of SFA is correlated to a significant minor risk of cardiovascular disease (CDV) and can lead to health benefits [21]. Fatty acids are a minor nutritional parameter of snail meat [4,9,10]. However, all of the raw snail samples examined in this work showed a low SFA content, in accordance to what was reported by Szkucik et al. [10] in farmed *C. aspersum* samples from Poland, but in contrast to what was found in wild *Helix pomatia* samples from Southern Turkey [9].

According to the literature, the factors of critical importance for the snail meat FA profile are the snail genus and its collection site. Interspecies differences in fatty acid composition were also confirmed in this work for the MUFA contents. In particular, the *T. pisana* raw meat samples showed C20:5 contents up to 16 times higher than *C. aspersum* and *E. vermiculata* samples; these differences could be due to the different ecological aspects of the species examined. It is well known that *T. pisana* is an agricultural pest in many parts of the world [21,22], feeding on a wide range of agricultural plants, including cereals with high UFA contents [14,23]. Differently from *T. pisana*, *C. aspersum* and *E. vermiculata* appear to be selective polyphagous organisms, preferring plants of the Poaceae family [12,24,25]. Other studies have confirmed how the feeding regimen would affect the fatty acid composition [4], verifying significant variations of MUFA contents related to the increase of soybean oil as feed supply in reared *C. aspersum* samples. Feeds that include corn, sunflower, or soybean rich in ω6 acids were shown to increase the content of these FAs in meat.

Nevertheless, the wild snail samples examined in this work showed high contents of UFA, constituting up to 79% of the total fatty acids. The level of PUFA in edible snails was found to be higher than SFA and MUFA, according to what was found in *Helix lucorum* and *Limax flavus* [26]. Our results are contrary to what was reported by Ekin et al. [27] in free-living *Melanopsis praemorsa* snails of Anatolia (Turkey) that showed a lower level of UFA and higher SFA contents. Essential fatty acids such as linoleic acid, α linolenic acid, docosahexaenoic acid (DHA), and eicosapentaenoic acid (EPA) were determined. These fatty acids show protection effects against cardiovascular disease [28,29]; however, the current intakes of EPA and DHA in European populations appear to be below the recommended daily allowance (RDA) [29].

The thermal processing of the snail samples analyzed determined an overall reduction of PUFA levels and a species-specific variation of MUFA and SFA contents, in contrast to what was found by Szkucik et al. [10] in farmed *C. aspersum* samples from Poland, verifying a significant increase of the SFA levels. The PUFA amounts of the samples analyzed decreased up to 7.98%, with a significant decrease of the C 20:5 contents in *T. pisana* samples. Nevertheless, PUFA remained the principal component, accounting for 44% of the total fatty acid contents in all of the species examined.

Among the MUFA, oleic acid (C18:1) remained the most abundant fatty acid of all of the snails species examined, even after heat processing.

Regarding the SFA contents, our results appear to comply with what was reported by Purwaningsih et al. [11] in mollusk muscles, showing that the SFA composition depends primarily on the snail species, rather than the way of cooking. The heat treatment of *T. pisana* samples determined a decrease of ɷ3/ɷ6 ratio from 0.58 to 0.3, reaching a value lower than the minimal ratio recommended by the WHO [30]. However, the heat treatment allowed obtaining a total degradation of toxic fatty acid as the erucic acid. Animal tests showed that the ingestion of oils containing erucic acid could lead to a heart disease called myocardial lipidosis. Other potential effects observed in animals (changes in liver, kidney, and skeletal muscle weight) occur at slightly higher doses.

Contrary to what was found by Szkucik et al. [10], the *C. aspersum* samples examined in this work showed a considerable decrease of the relative amounts of SFA after heat treatment, favoring an increase of the relative amounts of MUFA. The *E. vermiculata* samples showed a similar behavior of *C. aspersum* samples after heat treatment, showing a decrease of 8.72% for SFA and 4.66% for PUFA, and an increase of the amounts of MUFA. A reduced PUFA content could be caused by the autoxidation mechanisms initiated by temperature rise in meat during it is cooking [10,31]. Furthermore, these modifications appear to be related to the process temperature, the cooking time, and the internal temperature reached by the meat [31,32,33,34].

Principal component analysis allowed to depict the significant sources of variability of the dataset analyzed using two Principal Components (PCs), which showed a clear separation of the land snail samples according to species and heat treatment. The high percentage of variance explained by PC1 (93.89%) reflects the fact that the investigated variables were highly correlated, showing a general decrease due to heat treatment; this variation was much more pronounced for *T. pisana* than for the species *C. aspersum* and *E. vermiculata*. The highest variation after heat treatment common to all the three species was related to PUFA, MUFA, oleic acid, and linoleic acid.

## 5. Conclusions

To the best of our knowledge, this work reports, for the first time, the fatty acid composition of *T. pisana* samples and their variation as a result of heat processing, and the first time of fatty acid composition in *E. vermiculata, T. pisana,* and *C. aspersum* collected in Sicily (Southern Italy). The results showed a species-specific variation of FA contents in the land snails samples examined after boiling, showing the highest UFA decrease in *T. pisana* samples.

The results demonstrate that the land snail species examined could be a good source of MUFA and PUFA and their contents are species-specific. Boiling could be an adequate cooking procedure for land snail consumption according to retained nutritional and healthy criteria (PUFA contents and ω-6/ω-3 ratio). Furthermore, the boiling process can safeguard consumers against potentially pathogenic microorganisms. Given that boiling losses seem to be related to cooking time and temperature, further studies are needed to find the best cooking condition in order to preserve the best nutritional composition criteria of land snails.

## Figures and Tables

**Table 1:** Fatty acids contents (mean ± SD; g/100 g FA) in fat extracted from the land snails species examined (*n* = 3 replicates of each pool of samples). SFA = Saturated fatty acids, MUFA = monounsatured fatty acids, PUFA = polyunsaturated fatty acids.

Fatty Acid	*T. pisana* Raw	*T. pisana* Boiled	*C. aspersum* Raw	*C. aspersum* Boiled	*E. vermiculata* Raw	*E. vermiculata* Boiled
Myristic (C_14:0_)	0.73 ± 0.01	1.06 ± 0.01	0.76 ± 0.08	0.59 ± 0.03	0.81 ± 0.01	0.63 ± 0.00
Palmitic (C_16:0_)	12.63 ± 0.04	15.75 ± 0.16	16.02 ± 0.27	13.2 4 ± 0.36	14.63 ± 0.13	13.31 ± 0.00
Margaric (C_17:0_)	1.02 ± 0.01	1.22 ± 0.07	1.13 ± 0.05	1.35 ± 0.07	1.36 ± 0.07	1.04 ± 0.03
Stearic (C_18:0_)	5.41 ± 0.08	6.31 ± 0.96	7.72 ± 0.2	7.24 ± 0.14	7.66 ± 0.03	7.59 ± 0.01
Arachidic (C_20:0_)	0.63 ± 0.00	0.69 ± 0.04	0.71 ± 0.03	0.39 ± 0.02	0.81 ± 0.02	0.37 ± 0.00
Behenic (C_22:0_)	0.31 ± 0.00	0.29 ± 0.01	0.64 ± 0.04	0.19 ± 0.00	0.31 ± 0.02	0.39 ± 0.00
∑SFA	20.72	25.32	26.97	23.01	25.58	23.35
Myristoleic (C_14:1_)	0.53 ± 0.00	0.59 ± 0.02	0.52 ± 0.03	0.65 ± 0.03	0.23 ± 0.01	0.58 ± 0.02
Palmitoleic (C_16:1_)	0.50 ± 0.02	0.27 ± 0.05	0.32 ± 0.08	1.35 ± 0.06	0.40 ± 0.04	0.37 ± 0.01
Eptadecenoic (C_17:1_)	0.52 ± 0.01	0.54 ± 0.04	0.81 ± 0.04	1.08 ± 0.04	1.01 ± 0.03	1.10 ± 0.00
Eicosenoic (C_20:1_)	0.37 ± 0.00	0.26 ± 0.03	0.37 ± 0.04	0.50 ± 0.02	0.17 ± 0.05	0.48 ± 0.00
Erucic (C_22:1_)	0.52 ± 0.00	-	-	-	-	-
Oleic (C_18:1ω:9_)	28.83 ± 0.08	28.83 ± 0.46	23.79 ± 1.72	29.95 ± 0.46	26.03 ± 0.63	29.71 ± 0.07
∑MUFA	31.27	30.49	25.81	33.53	27.86	32.24
Linoleic (C_18:2 ω6_)	18.78 ± 0.02	21.35 ± 0.10	22.15 ± 0.13	19.07 ± 0.16	21.94 ± 0.13	19.20 ± 0.02
Linolenic (C_18:3 ω3_)	7.64 ± 0.02	8.87 ± 0.16	15.78 ± 0.09	15.14 ± 0.20	12.40 ± 0.41	15.53 ± 0.00
Eicosadienoic (C_20:2_)	5.29 ± 0.02	5.44 ± 0.04	3.98 ± 0.02	4.64 ± 0.02	4.21 ± 0.40	4.69 ± 0.00
Arachidonic (C_20:4_)	6.28 ± 0.08	7.17 ± 0.19	4.26 ± 0.20	4.18 ± 0.01	6.40 ± 0.52	4.27 ± 0.03
Eicosapentaenoic (C_20:5_)	9.85 ± 0.01	1.15 ± 0.51	0.62 ± 0.07	0.40 ± 0.05	1.30 ± 0.06	0.43 ± 0.01
Docosahexaenoic (C_22:6_)	0.18 ± 0.01	0.21 ± 0.00	0.43 ± 0.01	0.13 ± 0.00	0.33 ± 0.03	0.29 ± 0.01
∑PUFA	48.10	44.19	47.22	43.56	46.56	44.41
ω3/ω6	0.58	0.30	0.55	0.56	0.43	0.58
PUFA/SFA	2.32	1.75	1.75	1.89	1.82	1.90

## References

[1] Pellati R. Lumache e Consumi. http://www.fosan.it/notiziario/31_lumache_e_consumi.html.

[2] Cicero A., Giangrosso G., Cammilleri G., Macaluso A., Currò V., Galuppo L., Vargetto D., Vicari D., Ferrantelli V. (2015). Microbiological and Chemical Analysis of Land Snails Commercialised in Sicily. Ital. J. Food Saf..

[3] Milinsk M.C., das Graças Padre R., Hayashi C., de Oliveira C.C., Visentainer J.V., de Souza N.E., Matsushita M. (2006). Effects of feed protein and lipid contents on fatty acid profile of snail (Helix aspersa maxima) meat. J. Food Compos. Anal..

[4] Alasalvar C., Pelvan E., Topal B. (2010). Effects of roasting on oil and fatty acid composition of Turkish hazelnut varieties (*Corylus avellana* L.). Int. J. Food Sci. Nutr..

[5] Ayas D., Ozogul Y., Ozogul İ., Uçar Y. (2012). The effects of season and sex on fat, fatty acids and protein contents of Sepia officinalis in the northeastern Mediterranean Sea. Int. J. Food Sci. Nutr..

[6] Özogul Y., Özogul F., Çiçek E., Polat A., Kuley E. (2009). Fat content and fatty acid compositions of 34 marine water fish species from the Mediterranean Sea. Int. J. Food Sci. Nutr..

[7] Tangolar S.G., Özoğul Y., Tangolar S., Torun A. (2009). Evaluation of fatty acid profiles and mineral content of grape seed oil of some grape genotypes. Int. J. Food Sci. Nutr..

[8] Tokuşoğlu Ö. (2006). The quality properties and saturated and unsaturated fatty acid profiles of quail egg: The alterations of fatty acids with process effects. Int. J. Food Sci. Nutr..

[9] Özogul Y., Özogul F., Olgunoglu A.I. (2005). Fatty acid profile and mineral content of the wild snail (Helix pomatia) from the region of the south of the Turkey. Eur. Food Res. Technol..

[10] Szkucik K., Ziomek M., Paszkiewicz W., Drozd Ł., Gondek M., Knysz P. (2018). Fatty acid profile in fat obtained from edible part of land snails harvested in Poland. J. Vet. Res..

[11] Purwaningsih S., Suseno S.H., Salamah E., Mulyaningtyas J.R., Dewi Y.P. (2015). Effect of boiling and steaming on the profile fatty acids and cholesterol in muscle tissue of molluscs. Int. Food Res. J..

[12] Lazaridou-Dimitriadou M., Kattoulas M.E. (1991). Energy flux in a natural population of the land snail *Eobania vermiculata* (*Müller*) (*Gastropoda*: *Pulmonata*: *Stylommatophora*) in Greece. Can. J. Zool..

[13] Nicolai A. (2010). The Impact of Diet Treatment on Reproduction and Thermophysiological Processes in the Land Snails Cornu Aspersum and Helix Pomatia. Ph.D. Thesis.

[14] Odendaal L.J., Haupt T.M., Griffiths C.L. (2008). The alien invasive land snail Theba pisana in the West Coast National Park: Is there cause for concern?. Koedoe.

[15] Bertino E., Giribaldi M., Baro C., Giancotti V., Pazzi M., Peila C., Tonetto P., Arslanoglu S., Moro G.E., Cavallarin L. (2013). Effect of prolonged refrigeration on the lipid profile, lipase activity, and oxidative status of human milk. J. Pediatr. Gastroenterol. Nutr..

[16] Taverniers I., De Loose M., Van Bockstaele E. (2004). Trends in quality in the analytical laboratory. I. Traceability and measurement uncertainty of analytical results. TrAC Trends Anal. Chem..

[17] Pantano L., Cascio G.L., Alongi A., Cammilleri G., Vella A., Macaluso A., Cicero N., Migliazzo A., Ferrantelli V. (2016). Fatty acids determination in Bronte pistachios by gas chromatographic method. Nat. Prod. Res..

[18] Raimondi S., Luciani R., Sirangelo T.M., Amaretti A., Leonardi A., Ulrici A., Foca G., D’Auria G., Moya A., Zuliani V. (2019). Microbiota of sliced cooked ham packaged in modified atmosphere throughout the shelf life: Microbiota of sliced cooked ham in MAP. Int. J. Food Microbiol..

[19] van den Berg R.A., Hoefsloot H.C., Westerhuis J.A., Smilde A.K., van der Werf M.J. (2006). Centering, scaling, and transformations: Improving the biological information content of metabolomics data. BMC Genom..

[20] Ekin İ. (2015). A comparative study on fatty acid content of main organs and lipid classes of land snails *Assyriella escheriana* and *Assyriella guttata* distributed in southeastern Anatolia. Ital. J. Food Sci..

[21] Baker G.H. (1986). The Biology and Control of White Snails (*Mollusca*: *Helicidae*), Introduced Pests in Australia.

[22] Baker G.H. (1989). Damage, Population Dynamics, Movement and Control of Pest Helicid Snails in Southern Australia.

[23] Cowie R.H. (1984). The Life-Cycle and Productivity of the Land Snail Theba pisana (*Mollusca*: *Helicidae*). J. Anim. Ecol..

[24] Chevalier L., Coz M., Charrier M. (2003). Influence of inorganic compounds on food selection by the brown garden snail *Cornu aspersum* (*Müller*) (*Gastropoda*: *Pulmonata*). Malacologia.

[25] Chevalier L., Desbuquois C., Le Lannic J., Charrier M. (2001). Poaceae in the natural diet of the snail Helix aspersa *Müller* (*Gastropoda*, *Pulmonata*). Comptes Rendus de l’Académie des Sci.-Series III-Sci. de la Vie.

[26] Ekin I., Şeşen R. (2017). Investigation of the Fatty Acid Contents of Edible Snails Helix lucorum, Eobania vermiculata and Non-Edible Slug *Limax flavus*. Rec. Nat. Prod..

[27] Ekin İ., Başhan M., Şeşen R. (2011). Possible seasonal variation of the fatty acid composition from Melanopsis praemorsa (L., 1758) (*Gastropoda*: *Prosobranchia*), from southeast Anatolia, Turkey. Turk. J. Biol..

[28] Harper P.C. (1976). Breeding biology of the fairy prion (*Pachyptila turtur*) at the Poor Knights Islands, New Zealand. N. Z. J. Zool..

[29] Givens D.I., Gibbs R.A. (2008). Current intakes of EPA and DHA in European populations and the potential of animal-derived foods to increase them: Symposium on ‘How can the n-3 content of the diet be improved?’. Proc. Nutr. Soc..

[30] WHO, Expert Consultation on Diet, Nutrition, and the Prevention of Chronic Diseases, Weltgesundheitsorganisation, FAO (2003). Diet, Nutrition, and the Prevention of Chronic Diseases: Report of A WHO-FAO Expert Consultation; [Joint WHO-FAO Expert Consultation on Diet, Nutrition, and the Prevention of Chronic Diseases, 2002, Geneva, Switzerland].

[31] Weber J., Bochi V.C., Ribeiro C.P., Victório A.D.M., Emanuelli T. (2008). Effect of different cooking methods on the oxidation, proximate and fatty acid composition of silver catfish (*Rhamdia quelen*) fillets. Food Chem..

[32] Alfaia C.M.M., Alves S.P., Lopes A.F., Fernandes M.J.E., Costa A.S.H., Fontes C.M.G.A., Castro M.L.F., Bessa R.J.B., Prates J.A.M. (2010). Effect of cooking methods on fatty acids, conjugated isomers of linoleic acid and nutritional quality of beef intramuscular fat. Meat Sci..

[33] Domínguez R., Gómez M., Fonseca S., Lorenzo J.M. (2014). Influence of thermal treatment on formation of volatile compounds, cooking loss and lipid oxidation in foal meat. LWT-Food Sci. Technol..

[34] Rasinska E., Rutkowska J., Czarniecka-Skubina E., Tambor K. (2019). Effects of cooking methods on changes in fatty acids contents, lipid oxidation and volatile compounds of rabbit meat. LWT.

